# Eye-movements deficits in schizophrenia: A metanalysis of evidence

**DOI:** 10.1192/j.eurpsy.2021.1372

**Published:** 2021-08-13

**Authors:** M. Orsoni, S. Dal Col, C. Ruscelli, R. Sant’Angelo, M. Benassi

**Affiliations:** 1 Department Of Psychology, University of Bologna, Cesena, Italy; 2 Mental Health Department, AUSL ROMAGNA, Cesena, Italy

**Keywords:** Eye-movements, schizophrénia, Antisaccadic error, biomarker for schizophrenia

## Abstract

**Introduction:**

Although eye-movement disorders are one of the most replicated deficits in the psychiatric literature, the strong heterogeneity of results is still an unexplained issue that could be effectively addressed with a quantitative review of evidence.

**Objectives:**

For this reason, a large-scale metanalytic study comprising more than 200 studies was conducted to analyse the presence of eye-movement deficits in schizophrenia patients, as compared to healthy controls.

**Methods:**

To this aim, saccadic eye movements were grouped based on the type of task required (e.g., standard, predictive) and the quantification method used (e.g., number, duration, amplitude). For each sub-group separate meta-analysis were computed. Cohen’s d was used as measure of effect size. Risk of bias within and between studies and heterogeneity were also analysed.

**Results:**

indicated low Cohen’s d with the exception of the number of correct antisaccades – where schizophrenia patients reportedless correct anti-saccadesthan healthy controls - and antisaccades error rate – where schizophrenia patients reported a higher number of errors than healthy controls.

**Conclusions:**

Antisaccades emerged as better suited to differentiate between patients and healthy controls, thus making them the most promising candidate as a possible biomarker for schizophrenia.

